# In silico prediction of heme binding in proteins

**DOI:** 10.1016/j.jbc.2024.107250

**Published:** 2024-04-02

**Authors:** Noa A. Marson, Andrea E. Gallio, Suman K. Mandal, Roman A. Laskowski, Emma L. Raven

**Affiliations:** 1 School of Chemistry, University of Bristol, Bristol, UK; 2 European Bioinformatics Institute (EMBL-EBI), European Molecular Biology Laboratory, Wellcome Trust Genome Campus, Cambridge, UK

**Keywords:** heme, regulation, heme binding, ProFunc, ligand

## Abstract

The process of heme binding to a protein is prevalent in almost all forms of life to control many important biological properties, such as O_2_-binding, electron transfer, gas sensing or to build catalytic power. In these cases, heme typically binds tightly (irreversibly) to a protein in a discrete heme binding pocket, with one or two heme ligands provided most commonly to the heme iron by His, Cys or Tyr residues. Heme binding can also be used as a regulatory mechanism, for example in transcriptional regulation or ion channel control. When used as a regulator, heme binds more weakly, with different heme ligations and without the need for a discrete heme pocket. This makes the characterization of heme regulatory proteins difficult, and new approaches are needed to predict and understand the heme-protein interactions. We apply a modified version of the ProFunc bioinformatics tool to identify heme-binding sites in a test set of heme-dependent regulatory proteins taken from the Protein Data Bank and AlphaFold models. The potential heme binding sites identified can be easily visualized in PyMol and, if necessary, optimized with RosettaDOCK. We demonstrate that the methodology can be used to identify heme-binding sites in proteins, including in cases where there is no crystal structure available, but the methodology is more accurate when the quality of the structural information is high. The ProFunc tool, with the modification used in this work, is publicly available at https://www.ebi.ac.uk/thornton-srv/databases/profunc and can be readily adopted for the examination of new heme binding targets.

The hydrophobic complex formed between iron and protoporphyrin IX complex, commonly referred to as heme, is at the same time both essential for life and destructively cytotoxic. In cells, heme is thus presumed to be under tight regulatory control (1). The more well-understood functions of heme include oxygen storage and transport (the globins) (2, 3), electron transfer (the cytochromes) (4), oxygen activation and catalysis (the P450s, peroxidases, dioxygenases, and many other heme enzymes) (5, 6), the synthesis of cell signaling gases such as nitric oxide and carbon monoxide (nitric oxide synthase and heme oxygenase) (7, 8), and heme-based gas sensor proteins (soluble guanylate cyclase) (9, 10, 11).

It has been widely assumed that heme binds to the majority of heme proteins, such as the globins and most of the catalytic heme enzymes, with high or relatively high affinity and, therefore, irreversibly. But the logic of heme also acting as a biological regulator – for which there is now ample evidence (12, 13, 14, 15, 16, 17, 18, 19, 20) – does not fit with this concept, as heme may need to bind transiently and reversibly in order to exert any regulatory role. A number of heme-regulated cellular functions have now been uncovered where low affinity, presumed to be reversible, heme binding more sensibly accounts for activation/inactivation of cellular pathways (21, 22, 23, 24, 25, 26). This substantially extends the spectrum of known heme-binding affinities in heme proteins, as shown in Figure 1. In this context, it has been noted (27) that numerous heme proteins might well exist as a mixture of heme-bound and heme-unbound (*apo*) forms, and there is evidence for this in several cases (27, 28).

**Figure 1:**
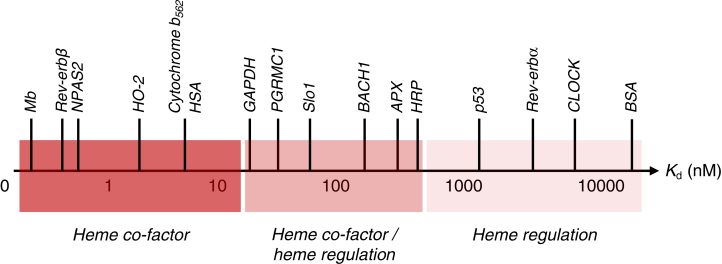
**Depiction of heme binding affinities (*K***_**d**_**, nM) for a selection of reported heme-binding proteins.** Affinity decreases from *left* to *right* (represented by the *red shading*). In this depiction, heme binding affinities have been categorized according to the different roles of heme (*i.e.* heme as a simple co-factor, *left*, or a regulator, *right*). The *middle box* designated ‘heme co-factor/heme regulation’ represents a region of heme binding affinities that cannot be assigned to either category alone. Reported heme affinities are taken from the literature (Table S1).

Heme binding to most proteins typically occurs through the coordination of the heme iron by axial ligands provided by the protein. Histidine is commonly employed, although methionine, cysteine, tyrosine, and even proline ligands are known (29). Heme binding pockets in these cases generally comprise ensembles of protein side chains that define a microenvironment tuned to accommodate the hydrophobicity and reactivity of the prosthetic group. There are thousands of published heme protein structures now available that conform to this model of heme binding.

The picture is different when heme binds in a regulatory capacity. In these cases, heme binds to proteins using different interactions and, in the cases identified so far, with lower affinity (30). These low-affinity interactions mean that structural information for such heme binding is, with a few exceptions, not available. For proteins not in the Protein Data Bank (PDB), structural prediction approaches based on artificial intelligence, such as the AlphaFold database (31, 32, 33), can provide structural information. However, AlphaFold cannot predict whether heme (or any other ligand) binds to the protein (34). Since new heme regulatory proteins have been frequently identified in recent years, there is a need for methods that can identify these transient heme binding interactions.

In this paper, we use the template-based ligand searches of ProFunc (35) to predict the heme binding locations of a number of regulatory proteins that have been reported to interact with heme. We anticipate this methodology will be useful in the future as new heme targets emerge in areas of heme-dependent cell signaling, heme trafficking, and the role of heme proteins in human disease.

## Results

The webserver ProFunc accepts structural files in PDB format (*e.g.* a crystal structure or AlphaFold model) as input and uses a number of sequence- and structure-based methods to help identify the likely function of the submitted protein (35). One of the structure-based searches it performs is against a large dataset of ligand-binding templates. These consist of the 3D conformations of residue triplets interacting with specific ligands in the PDB. For any ligand, there may be many templates, and many different templates may find a match in the query protein. All hits are scored according to the similarity between the local environment around the template in the source PDB file and that in the submitted structure. The highest scores come from structural homologs. Here we are interested in potential heme binding sites, including low-scoring matches to analogous binding sites which are difficult to find. Therefore, the ProFunc server was modified to allow a search against just heme-related templates and to keep even low-scoring hits for subsequent manual checking. This modification is available in ProFunc and can be extended to other molecules of interest (*e.g.* NAD, ATP, Zn) on request.

**Figure 2:**
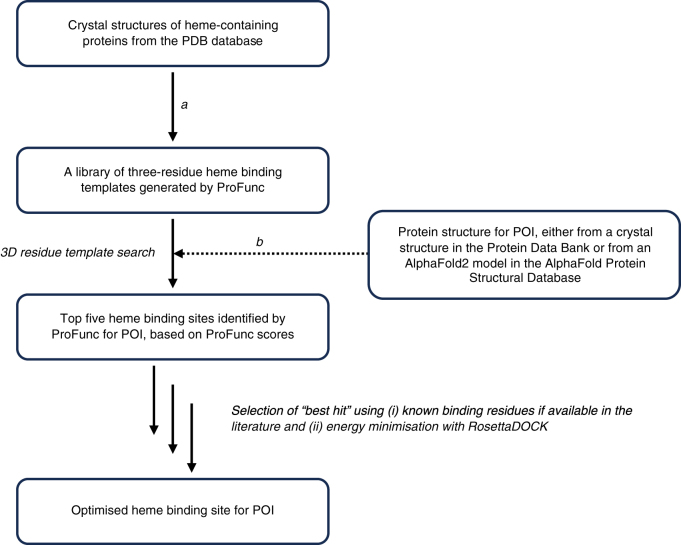
**Workflow showing the methodology used to obtain predicted heme binding sites from ProFunc.** Heme templates in ProFunc were created from all of the heme-containing structures in the PDB (which numbered 5712 when the templates were generated, step *a*). Of these structures, we did not identify whether they bind heme tightly or weakly (see also Table S2). In step *b*, crystal structures from the Protein Data Bank (shown as *orange* structures in Figs. 4 and S3*A*) were used for CLOCK and GAPDH. AlphaFold models obtained from the AlphaFold Protein Structure Database (shown as magenta structures in Figs. 3 and 5, S2, S3*B* and S4) were used for HO-2, PGRMC1, Rev-erbβ, STEAP1, ALAS-1, BACH1, FRP1, NPAS2, PER2 and Rev-erbα. POI, protein of interest.

The modified version of ProFunc was run on a test set of known or putative heme-binding proteins using PDB structures, where available, or AlphaFold models, where not (Fig. 2). The proteins included transcriptional regulators (BACH1, Rev-erbα) (36, 37, 38, 39), proteins involved in the regulation of circadian rhythms (CLOCK, NPAS2, PER2) (40, 41, 42), heme synthesis and degradation proteins (ALAS-1, HO-2) (43, 44, 45, 46, 47, 48, 49, 50, 51), and others known to bind heme in a regulatory capacity (FRP1, GAPDH, PGRMC1, Rev-erbβ, STEAP1) (52, 53, 54, 55, 56, 57, 58, 59). Table 1 lists the proteins, their PDB codes, and UniProt accessions, together with their proposed functions. For the purposes of the analyses presented in this article, these heme-binding proteins were categorized as follows: (i) proteins for which a heme-bound crystal structure is available in the PDB (HO-2, PGRMC1, Rev-erbβ, and STEAP1); (ii) proteins for which a crystal structure is available but without heme bound (GAPDH and CLOCK); (iii) proteins for which no crystal structure of the heme binding region is available (ALAS-1, BACH1, FRP1, NPAS2, PER2, and Rev-erbα).

**Table 1:** List of known or putative heme-binding proteins used in this work, with PDB codes, UniProt IDs and proposed functions shown

Protein name (abbreviation)	Species	PDB	UniProt ID	Proposed function	ProFunc prediction consistent with literature?
Heme oxygenase 2 (HO-2)^a^	*Homo sapiens*	2QPP	P30519	Heme degradation	Yes
Membrane-associated progesterone receptor component 1 (PGRMC1)^a^	*Homo sapiens*	4X8Y	O00264	Role in heme homeostasis	Yes
Nuclear receptor subfamily 1 group D member 2 (Rev-erbβ)^a^	*Homo sapiens*	6WMQ	Q14995	Heme-dependent transcriptional regulation	Yes
Six-transmembrane epithelial antigen of prostate 1 (STEAP1)^a^	*Homo sapiens*	6Y9B	Q9UHE8	Metalloreductase	Yes
Circadian locomoter output cycles protein kaput (CLOCK)^b^	*Homo sapiens*	6QPJ^c^	O15516	Transcriptional activator involved in circadian clock	Yes
Glyceraldehyde-3-phosphate dehydrogenase (GAPDH)^b^	*Homo sapiens*	1ZNQ	P04406	Moonlights as a heme chaperone	No
5-aminolevulinate synthase, non-specific, mitochondrial (ALAS-1)^d^	*Homo sapiens*	n.a.	P13196	Heme synthetic pathway	No
Transcription regulator protein BACH1 (BACH1)^d^	*Homo sapiens*	2IHC^e^	O14867	Transcriptional regulator	No
Frp1p (FRP1)^d^	*Candida albicans*	n.a.	A0A1D8PKY2	Transmembrane protein	Yes^f^
Neuronal PAS domain-containing protein 2 (NPAS2)^d^	*Homo sapiens*	n.a.	Q99743	Transcriptional activator involved in circadian clock	Yes
Period circadian protein homolog 2 (PER2)^d^	*Homo sapiens*	6OF7^e^	O15055	Transcriptional repressor involved in circadian clock	No
Nuclear receptor subfamily 1 group D member 1 (Rev-erbα)^d^	*Homo sapiens*	3N00^e^	P20393	Nuclear receptor	Yes
Bovine serum albumin (BSA)	*Bos taurus*	3V03	P02769	Plasma protein	n.a.
Human serum albumin (HSA)	*Homo sapiens*	1N5U; 1O9X	P02768	Plasma protein; heme scavenging	n.a.

a Proteins for which a heme-bound crystal structure is available.

b Proteins for which a crystal structure is available, but without heme bound.

c This structure is for the PAS-A domain of the human CLOCK protein. There is also a full length structure for the mouse CLOCK protein, containing both PAS-A and PAS-B domains, but the human protein was used in this work (96).

d Proteins for which there is no crystal structure for the suspected heme binding region in the given species.

e This crystal structure is not the suspected heme-binding region.

f Not expected to be a regulatory heme binding site as likely to be involved in electron transport (97).

### Proteins for which a heme-bound crystal structure protein is available

**Figure 3:**
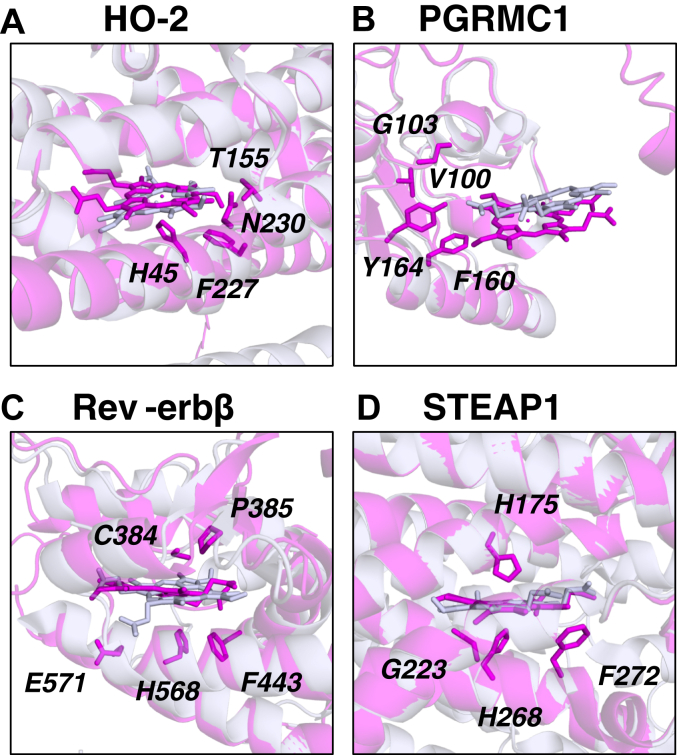
**Predicted heme-binding sites were obtained with ProFunc using AlphaFold models (shown in magenta) and optimized with RosettaDOCK for four proteins for which a heme-bound crystal structure is available in the PDB.** The heme-binding sites obtained in this way have been aligned with the heme-binding site found in the crystal structure (shown in *grey*) obtained from the PDB. The alignments of the predictions in AlphaFold models and heme-bound crystal structures are also shown for the full length of the proteins in Fig. S2. The proteins examined were (*A*) HO-2 (PDB code 2QPP) (51); (*B*) PGRMC1 (PDB code 4X8Y) (56); (*C*) Rev-erbβ (PDB code 6WMQ) (58); and (*D*) STEAP1 (PDB code 6Y9B) (59).

We first analyzed the predicted binding sites for four heme proteins with known heme binding sites (HO-2, PGRMC1, Rev-erbβ, and STEAP1). In this case, there are published heme-bound crystal structures available in the PDB (Table 1), providing reliable information on the location of the heme and the binding residues. In each case, the predicted heme binding site (obtained from ProFunc using the methodology shown in Fig. 2) and the known crystal structure from the PDB were aligned in PyMOL (60). This allowed assessment of the methodology as a prediction tool and, in particular, assessment of the reliability of predictions in cases where there is no crystal structure for the heme-bound protein (as below). In all cases where a heme-bound crystal structure is available, ProFunc successfully determined the region of the heme-binding site, Figure 3.

### Proteins for which a crystal structure is available but without heme bound

**Figure 4:**
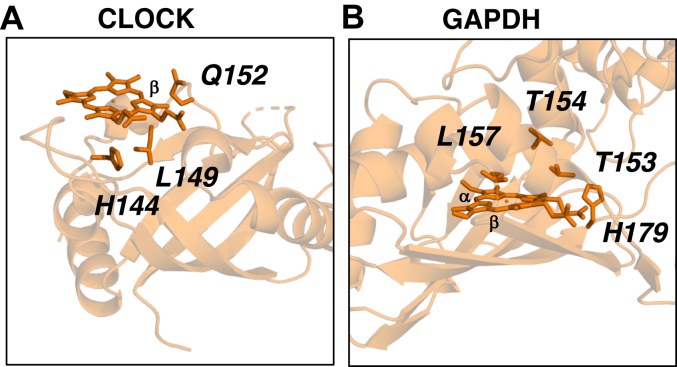
**Predicted heme binding sites obtained with ProFunc using *apo*-crystal structures (orange) from the PDBand optimized with RosettaDOCK.** The proteins examined were (*A*) CLOCK (PDB code 6QPJ) (62) and (*B*) GAPDH (PDB code 1ZNQ) (63).

We examined two proteins in the test set for which there are crystal structures of the *apo*-protein (*i.e.* without heme bound) available in the PDB – CLOCK and GAPDH, Figure 4. In these two cases, residues that have been identified as being involved in heme binding (61, 62) are present in the published crystal structures (62, 63) (which is not the case for the proteins discussed in the section below). ProFunc was used to identify the heme binding regions, and then RosettaDOCK was used to optimize these binding sites in the crystal structures from the PDB.

For CLOCK (PDB code 6QPJ), His144, Leu149, and Gly152 were identified by ProFunc as close to the heme binding region, although the heme binding location identified using the PAS-A domain for human CLOCK (PDB code 6QPJ) is on the surface and potentially unfeasible, Figure 4*A*. Following optimization by docking, the model shows that the distance from the Nδ of His144 to the Fe of the heme is 5.6 Å, Figure 4*A*. While this is not within bonding distance, it is nonetheless consistent with the identification of His144 as a heme-binding ligand (62). Leu149 and Gly152 are noted as interacting with the β-edge of heme (64). Using the AlphaFold model of human CLOCK, which contains both the PAS-A and PAS-B domains of the protein, the additional PAS-B domain occupies the space above the heme (as shown in Fig. S4). This demonstrates that the location of the heme binding site in Figure 4*A* is plausible.

For GAPDH (PDB code 1ZNQ), the ProFunc model shows Thr153 and Thr154 interacting with the propionate groups at the polar end of the heme molecule, and Leu157 interacting with the hydrophobic α/β edges of the heme, Figure 4*B*. His179, as identified by ProFunc, is identified close to the heme binding site and is a possible axial ligand. However, the orientation of His179, and the distance from the heme, is unsuitable for its ligation to the iron; modelling with RosettaDOCK confirmed this and showed that His179 is unlikely to be interacting with the heme iron (Nδ-Fe and Nε-Fe distances are 11.4 and 10.0 Å, Fig. S3). His179 in GAPDH has been ruled out as a heme ligand, based on mutagenesis work (61), which is in agreement with our analyses. His53 has been identified (61) as the most likely histidine residue in GAPDH, however, this was not identified by ProFunc.

### Proteins for which no crystal structure of the heme binding region is available

**Figure 5:**
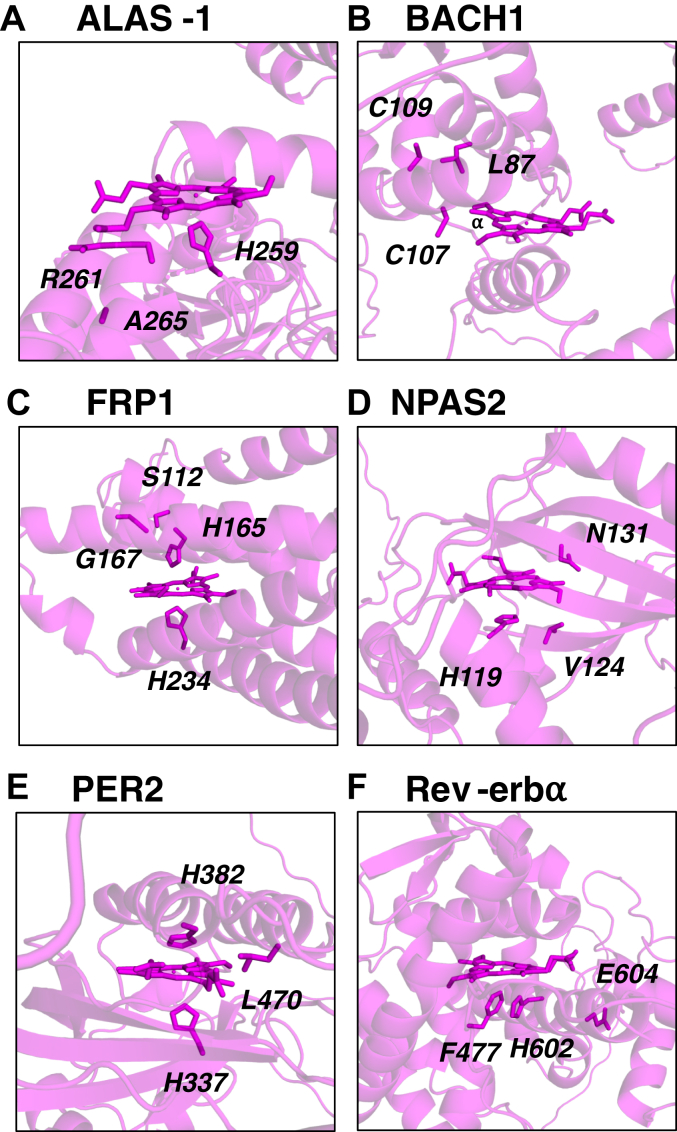
**Predicted heme binding sites obtained from ProFunc using AlphaFold modelsand optimized with RosettaDOCK.** The proteins examined were (*A*) ALAS1, (*B*) BACH1, (*C*) FRP1, (*D*) NPAS2, (*E*) PER2, and (*F*) Rev-erbα, and optimized with RosettaDOCK. There are no complete crystal structures for these proteins available in the PDB.

We examined a series of proteins for which either no crystal structures are available or only partial structures, Figure 5. These proteins are ALAS-1, BACH1, FRP1, NPAS2, PER2, and Rev-erbα, Table 1. Unlike CLOCK and GAPDH above, in this series of proteins the published crystal structures, if they exist, do not include the regions suspected to be involved in heme binding. In these cases, where crystal structure information is not available, a structural model (*e.g.* from AlphaFold) can be used as the input file for ProFunc (step *b* in Fig. 2). It is important to note that the confidence of AlphaFold models is variable, and this is given by a per-residue confidence metric (pLDDT), provided for each structure (65). These confidence levels are an important consideration in the following results.

The binding site identified by ProFunc in the ALAS1 model (UniProt ID P13196) consists of His259, Arg261, and Ala265, Figure 5*A*. His259 is identified as a possible axial ligand and is within a reasonable range (Nε-Fe distance is 4.50 Å). Arg261 interacts with one of the propionate groups. However, modeling with RosettaDOCK suggests that a heme interaction with Ala265 is unlikely. Heme is located between three α-helices in a well-structured region of the model and the propionate groups are exposed and pointing away from the protein. Mutagenesis studies have implicated a so-called CP motif in heme binding (Cys108-Pro109) in the human protein (46). However, this motif is in a region of low or very low confidence in the AlphaFold model, so was unlikely to be identified by ProFunc.

In the predicted model for the transcription factor BACH1 (UniProt ID O14867), Figure 5*B*, the identified heme binding residues are Cys107, Cys109, and Leu87. These residues are located on alpha helices and interact with the hydrophobic α-edge of the heme. BACH1 is thought to bind heme *via* one of several CP motifs located at Cys224, Cys301, Cys438, Cys464, Cys495, and Cys649 (66, 67). No CP motifs were identified in the ProFunc model (Cys107 and Cys109 are not part of CP motifs).

For the ferric reductase-related protein FRP1 (UniProt ID A0A1D8PKY2) (52, 68), the predicted binding site shows His165 and His234 acting as potential axial ligands to heme, Figure 5*C*. Ser112 and Gly167 were also identified as binding residues. Heme is shown to be buried within alpha helices in a region of high secondary structure. This is a convincing site for binding heme but whether this is a regulatory heme binding site is unclear at this stage (68).

NPAS2 (UniProt ID Q99743) is an important protein involved in the regulation of the circadian clock *via* its binding to DNA. In the AlphaFold model used here, His119, Val124, and Asn131 were identified as heme binding residues, Figure 5*D*. NPAS2 is known to bind heme *via* His/Cys or His/His ligation (40, 69) using His119 and Cys170/His171 as the likely ligands (30, 40, 70). While His119 is identified in the predicted model from ProFunc, Cys170, and His171 are too far away to be involved in binding, Fig. S3*B*.

In the case of PER2 (UniProt ID O15055), His337, His382, and Lys470 were identified by ProFunc as heme binding residues, Figure 5*E*. A CP motif (Cys841-Pro842) has previously been identified as important for heme binding (71); however, this is a region of very low confidence in the AlphaFold model, making it unlikely that ProFunc would successfully predict heme binding in this region.

The heme-binding residues identified by ProFunc for Rev-erbα (UniProt ID P20393) are Phe477, His602, and Glu604, all of which are located on α-helices, Figure 5*F*. His602 has previously been identified as a heme binding residue (38). The ProFunc model confirms that His602 is a suitable ligand and provides a plausible binding site for heme.

**Figure 6:**
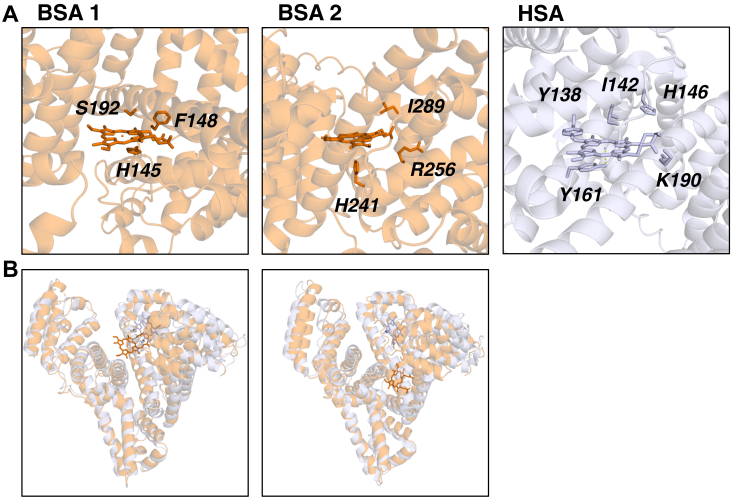
**The top two predicted heme binding sites for bovine serum albumin (BSA, PDB code****3V03****) were obtained by ProFunc and optimised by docking.***A*, the possible heme binding sites for BSA, with the residues identified by ProFunc labelled and the HSA heme binding site (PDB code 1N5U) shown for comparison. *B*, the full-length models of heme bound to BSA (*orange*) aligned with the heme-bound crystal structure for HSA (PDB code 1N5U, *grey*).

Finally, we tested the methodology on the intriguing case of human serum albumin (HSA, UniProt ID P02768) and bovine serum albumin (BSA, UniProt ID P02769). HSA and BSA are structurally very similar (Fig. 6, *A*–*C*), but have much different heme affinities (Fig. 1, Table S1). Crystal structures for heme bound to HSA are available (PDB ID 1N5U, 1O9X) (72, 73), Figure 6*C*. Using the methodology described in this paper, we identified two potential heme binding sites for BSA (using PDB code 3V03), Figure 6*A*. While each of these contains a possible His ligand at (3.5–4.6 Å from the iron), the sites are notably different from that in HSA. HSA contains a Tyr ligand (Tyr161) within 2.7 Å of the iron and two strong hydrogen bonds from His146 and Lys190 to the propionates (74), all of which are missing in the BSA models and which might account for the different affinities.

## Discussion

When heme is acting in a regulatory role, for which there is ample evidence (30), it typically binds to proteins with lower affinities, Figure 1. In these cases, heme binding pockets are likely to be more exposed to the solvent – on the surfaces of proteins or in between subunits (75, 76, 77) – rather than deeply buried in a specific heme binding pocket as has typically been observed for other heme proteins. The nature of these regulatory heme-protein binding interactions is therefore more difficult to establish experimentally, in part because the spectra of heme-binding regulatory proteins are different from the classical electron transfer/O_2_ binding/catalytic heme proteins. As these new roles for heme proteins are uncovered, the need to identify potentially transient heme binding sites becomes important so that they might, in future, be redesigned (for example in therapeutic interventions).

**Figure 7:**
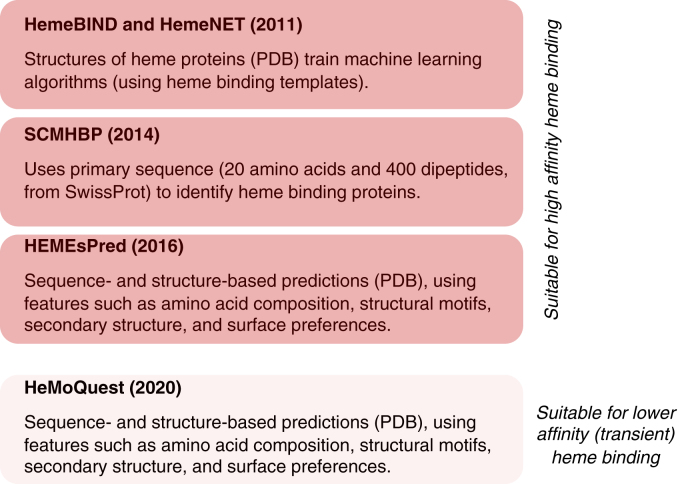
**An overview of the heme binding prediction toolsthat have been developed** (81, 82, 83, 84, 85, 94, 95)**.** The tools in the *red boxes* are better suited to predicting high-affinity heme binding whereas HeMoQuest (85) was developed to predict transient heme binding. These ‘heme-specific’ tools are a small subset of a much larger range of tools to predict protein-ligand interactions more generally. When using these tools, sequence information (*e.g.* a FASTA file) or structural data (*e.g.* a PDB file) must be submitted as an input, depending on the tool being used.

Proteomics methods (12, 78, 79, 80) have lately been employed to identify potential heme-binding proteins, but this approach gives no information on the location of the heme-binding site. In such cases, bioinformatics approaches can be helpful but have not been widely adopted in the field. A summary of bioinformatics tools developed specifically to assess heme binding is given in Figure 7. For example, HemeBIND and HemeNET (81, 82), SCMHBP (83), and HEMEsPRED (84) use a combination of structure- and sequence-based predictions. The output is a list of candidate residues that may bind to heme, sometimes with an associated probability (84). However, the results can be difficult to interpret and the heme-binding sites are not easily visualized. More recently, a machine learning tool named HeMoQuest (85) has been developed and is the first attempt at predicting heme binding sites within proteins that have much lower or transient heme binding affinities. Other tools include GalaxyDOCK2-HEME, a docking algorithm that has been developed specifically for heme binding (86); AWSEM-heme, which models the folding of heme proteins (87); and AlphaFill, specifically developed to add in small molecules and ions missing from AlphaFold models (88). However, for our subset of heme regulatory proteins, AlphaFill did not place heme into the AlphaFold models that were supplied. As a result, we could not use it for identifying heme-binding sites in these cases.

In this work, we apply the ProFunc tool to identify heme binding sites. ProFunc predicts the functions of proteins based on sequence or crystal structure information (from the PDB) and using various comparators, such as sequence searches, surface cleft analysis, residue conservation, and 3D template searches to identify functional motifs or structural homologs (35). AlphaFold models can also be used as the input information, Figure 2, which means that ProFunc can produce predictive information on heme binding even in cases where a crystal structure has not yet been determined. Unlike other heme-specific prediction tools available, ProFunc provides a 3D heme binding site that can be easily visualized (*e.g.* in PyMol) and, if relevant, further optimized— as we have done in this paper with RosettaDOCK. Optimizing the heme binding sites with RosettaDOCK was used to validate the likelihood of heme binding at the site predicted by ProFunc.

In the cases where a heme-bound crystal structure is already available (HO-2, PGRMC1, Rev-erbβ and STEAP1, Table 1), ProFunc was able to identify the heme-binding site, as expected - see alignments in Figure 3. These are trivial examples as the structures, or their homologs, were used by ProFunc to generate its heme-binding templates, but they serve as a useful control for the analyses where no crystal structures are available.

Two proteins (CLOCK and GAPDH) were tested for which there is a crystal structure of the suspected heme binding region, but no heme bound. For CLOCK, ProFunc correctly identified the proposed heme binding site (His144) from the literature (62). In this case, the crystal structure for the human protein used as the input file does not cover the full length of the protein (see Table 1 entry), so was repeated using the AlphaFold model and showed a more plausible heme binding site. However, for GAPDH, the result from ProFunc was only partially consistent with the literature (61)—His179 was correctly ruled out as a ligand, but His53, thought to be a ligand (61), was not identified by ProFunc.

The results for the final category—where there is no crystal structure for the heme-binding protein—were necessarily obtained using AlphaFold models. In the cases where heme binding to the protein is suspected to be through CP motifs, ProFunc was unable to identify the correct heme binding sites. CP motifs are a relatively recent category of heme-binding motif (30), and are not well-characterized in the PDB; consequently, our expectation is that ProFunc will be unable to identify these sites. Additionally, these CP motifs tend to be in regions of very low accuracy in the AlphaFold models, a challenge also noted recently in other work on heme-binding motifs (89).

Overall, the results of this work show that the methodology can identify potential heme-binding sites in some regulatory heme proteins and that the predictions are consistent with experimental evidence when there is reliable structural information available (*i.e.* a crystal structure or an AlphaFold model with high levels of confidence) for the protein of interest. The methodology is dependent on the quality of AlphaFold models supplied to ProFunc as input structures, and on the quality and availability of heme binding modes present in the PDB. It is also currently limited by the number of structures for heme-dependent regulatory proteins in the PDB - as this number increases, and particularly as AlphaFold develops, the reliability of ProFunc, and other predictive tools, will improve.

## Experimental procedures

### Heme binding site predictions

Heme binding sites for a test set of proteins were predicted using the bioinformatics tool ProFunc. ProFunc is accessed *via* its web server (https://www.ebi.ac.uk/thornton-srv/databases/profunc) and makes predictions of protein function based on sequence and structure information. In this case, the server was tailored to search specifically for heme-binding sites. This modification has been added to the server as an optional “ligand filter”. A step-by-step guide to using the server is provided in Fig. S1.

To search for possible heme binding sites, ProFunc (90) uses the 3-residue heme-binding templates it has derived from all proteins (5712 when the templates were generated) in the PDB containing heme (step *a* in Fig. 2). Of the 5712 structures that were used in step *a*, there is no simple way to identify whether they bind heme tightly or weakly. Each template is scanned against the input structure (step *b* in Fig. 2) and for each match, the two structures are aligned based on the match. A score is then assigned according to how many identical and similar residues within 10 Å of the template occur in equivalent 3D positions. In the standard ProFunc scoring function, this score is enhanced to bias for homologous proteins. However, for the heme searches, this bias is removed to allow potential sites to be identified from matches to non-homologous proteins, as such matches would not normally be reported.

### Visualisation of heme binding sites

The predicted heme-binding sites in the protein of interest generated in this way by ProFunc were visualized in PyMOL as PDB files (60), along with the heme binding template residues (above) to which the protein was most closely matched. The models were then manually screened in PyMOL for possible histidine and cysteine residues near the heme which could act as a ligand to the iron. In cases where a crystal structure for the heme-bound protein is already known, they were aligned with the output PDB file from ProFunc, to assess the reliability of the model. The overall workflow for this process is shown in Figure 2.

### Molecular modeling

Heme binding sites obtained from ProFunc were further optimized using RosettaDOCK. To dock the heme molecule in the predicted structure of each protein, the heme coordinate file (as a SDF file) was moved to the binding pocket, as predicted by ProFunc, of each protein PDB file using PyMOL. The protein PDB file was prepared for docking using RosettaScript file 'clean_pdb' and the ligand PDB file and parameters file were generated using RosettaScript file “molfile_to_params” (91). RosettaLigand docking was used to perform the docking on the prepared protein and heme files, with a ligand transform gridbox size of 7.0 Å^3^ and moving distance of 0.2 Å to generate 10 poses for the protein-heme complex (92, 93). The docking was performed based on the induced fit model where both ligand and protein active site residue sidechains are flexible.

## Data availability

All relevant data are available upon request from the corresponding author.

## Supporting information

This article contains supporting information (98, 99, 100, 101, 102, 103, 104, 105, 106, 107, 108).

## Conflict of interest

The authors declare that they have no known competing financial interests or personal relationships that could have appeared to influence the work reported in this paper.
