# Supplementary material for: In silico prediction of heme binding in proteins
Source: J Biol Chem. 2024 Apr 2;300(5):107250. doi: 10.1016/j.jbc.2024.107250 (PMC11101860; doi:10.1016/j.jbc.2024.107250)
Supplement: Supporting Information [file mmc1.pdf]

SUPPLEMENTARY INFORMATION

Table S1. List of proteins used in Figure 1, showing examples of reported heme binding affinities for the ferric forms of the proteins.

Protein	K_d (nM)	Ref.
Myoglobin (Mb)	0.000014	(98)
Nuclear receptor subfamily 1, group D, member 2 (Rev-erbβ)	0.1; 2000[a]	(18, 99)
Neuronal PAS domain protein 2 (NPAS2)	0.16	(100)
Heme oxygenase 2 (HO-2)	3.6	(49)
Cytochrome b_{562}	9	(101)
Human serum albumin (HSA)	9	(102)
Glyceraldehyde-3-phosphate dehydrogenase (GAPDH)	24; 150[a]	(61, 103)
Progesterone receptor membrane component 1 (PGRMC1)	50	(56)
Slo1	45 – 120	(104)
BTB domain and CNC homolog 1 (BACH1)	140	(36)
Ascorbate peroxidase (APX)	190, 360	(105, 106)
Horseradish peroxidase (HRP)	270	(106)
Tumor-suppressor protein p53 (p53)	1200	(107)
Nuclear receptor subfamily 1, group D, member 1 (Rev-erbα)	3000	(18)
Transcriptional regulatory protein associated with circadian rhythms (CLOCK)	4200	(62)
Bovine serum albumin (BSA)	20000	(108)

[a] Different values for the binding of heme to Rev-erbβ and GAPDH are reported.

Table S2. The nature of heme proteins in the PDB, used to generate the heme-binding templates described in this work.[a]

Category	Count
Oxidoreductase	4228
Oxygen binding/storage/transport	1481
Electron transport	327
Transport protein	214
Metal binding/transport	112
Photosynthesis	110
Signaling protein	84
Membrane protein	73
Lyase	42
Heme binding protein	40
Transferase	37
Peroxidase	23
Hydrolase	19
Biosynthetic protein	19
Unknown function	14
Iron binding/storage/transport	12
Transcription	10
Ligand binding protein	9
Plant protein	6
Photosynthetic reaction center	6
Lipid binding protein	6
Isomerase	6
De novo protein	6
Blood clotting	5
Apoptosis	5
Structural protein	4
Cytosolic protein	4
Protein binding	3
Nitric oxide transport	3
Fluorescent protein	3
Antibiotic	3
Proton transport	2
Plasma protein	2
Myeloperoxidase	2
Flavoprotein	2
DNA binding protein	2
Viral protein	1
Vasodilator	1
Translocase	1
Transcription regulator	1
Protein transport	1
Peptide binding protein	1
Ligase	1
Hormone	1
Endocytosis	1
Cytosolic protein inhibitor	1
Binding protein	1
Antimicrobial protein	1

[a] The categories have been extracted from the "header" for each protein in the PDB. Note that in the sub-set of 5,712 proteins from the PDB used to create the ProFunc templates, there is no simple way to identify whether they bind heme tightly or weakly. We counted the numbers of interactions each heme makes with its protein, but our analyses of these interactions give a wide range of values, and the structures with few interactions are those that we expect to have high heme affinity. Searching for weak heme binders was not attempted, as it requires verifying the role of each protein from the literature. The sub-set undoubtedly contains a few examples of low-affinity binders, but it is difficult to ascertain how many.

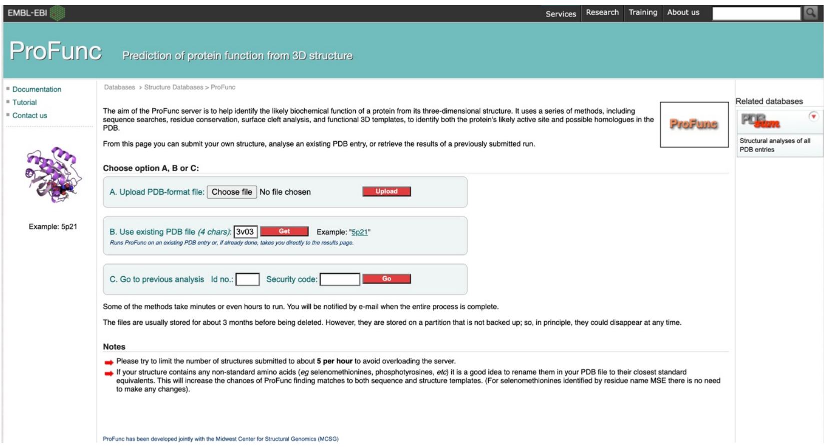

1. *Access https://www.ebi.ac.uk/thornton-srv/databases/profunc/ and (A) upload your PDB file for your protein of interest. This can be a crystal structure (e.g. obtained from the PDB database) or from the AlphaFold Protein Structure Database; or (B) use an existing PDB file by entering the PDB ID for the structure.*

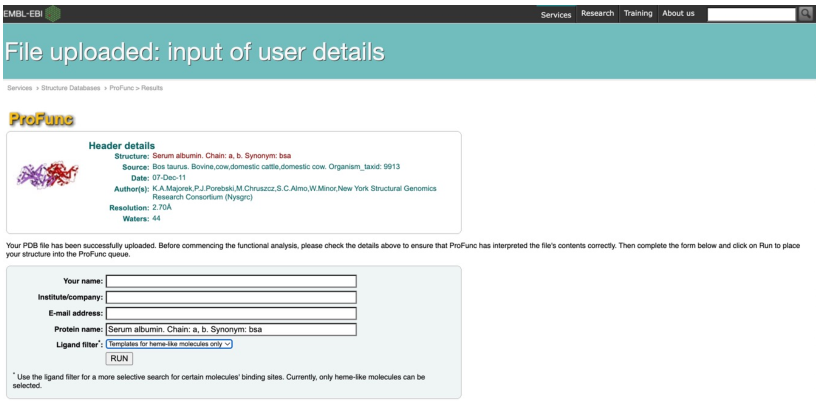

2. *Proceed to the next page and enter your details. At this point, you can select to search against "All ligand templates" or "Templates for heme-like molecules only". The former will search across all possible ligands whereas the latter will also scan your structure against heme binding templates using a modified scoring function. Click "run" and wait for an email from ProFunc with the results.*

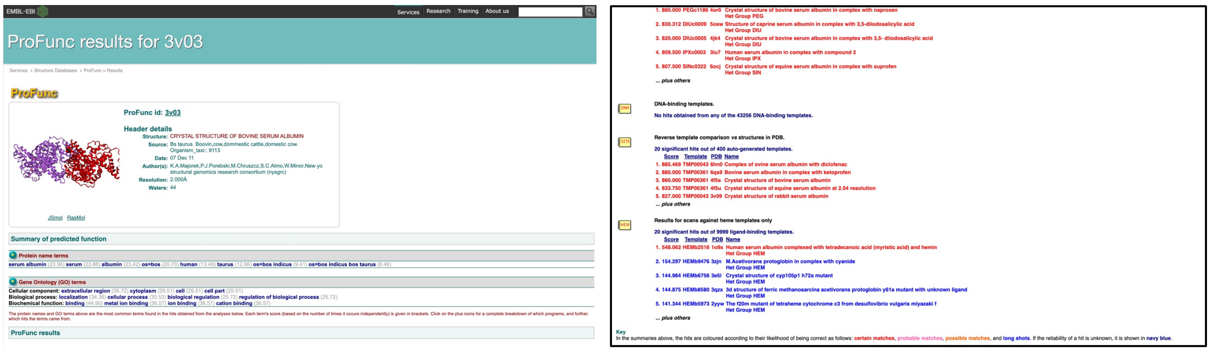

4. *Open the email to access the results for your file. Scroll to the bottom of the page to see the last category "HEM" which are the results for scans against heme templates only. Click on the yellow box to see more details.*

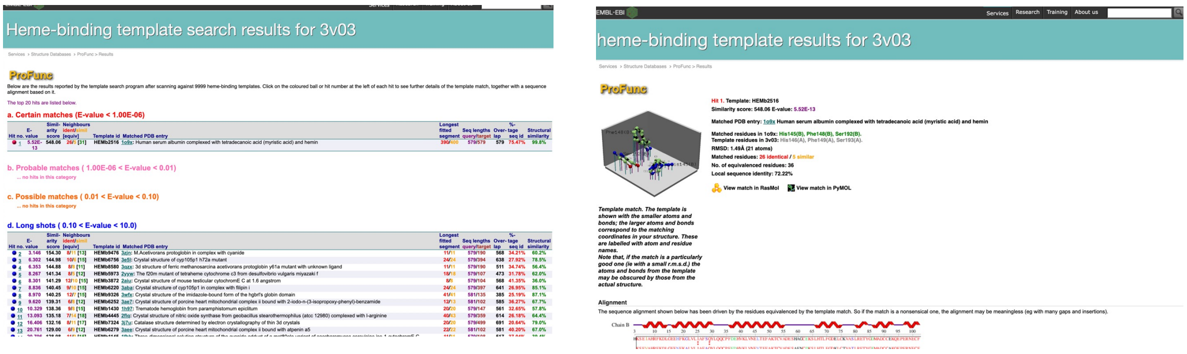

5. *The list of hits is categorised into "certain", "probable" and "possible" matches, and "long shots". The "long shots" may be matches to similar binding sites in analogues, rather than homologues. Clicking on the sphere at the left-hand side of each line will show more information about the match and provides a file to download the binding site in PyMol or RasMol.*

Fig. S1. Step-by-step guide demonstrating how to use ProFunc and the ligand filter specific for the identification of heme binding.

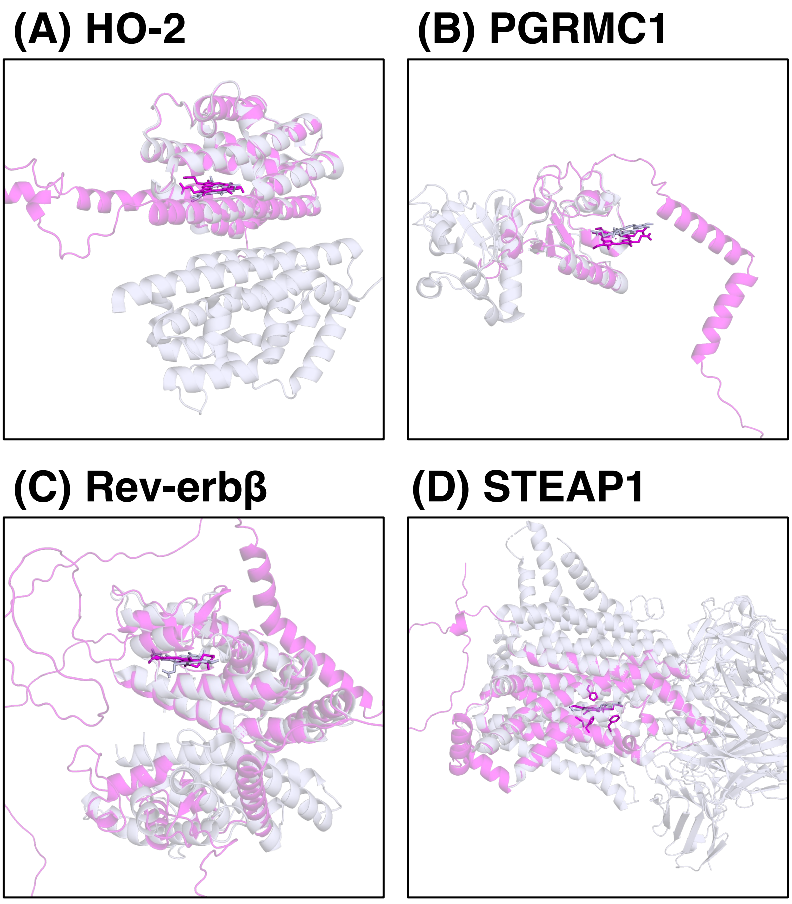

Fig. S2. Alignments of the heme-bound crystal structures obtained from the PDB (grey) with the predicted heme binding sites (obtained using ProFunc and optimised with RosettaDOCK) obtained from AlphaFold models (in magenta) for the four proteins shown in Fig. 3. This figure shows each protein in the same orientation as in Fig. 3 and highlights the close alignment of the heme-bound crystal structures with the AlphaFold models in the heme binding region.

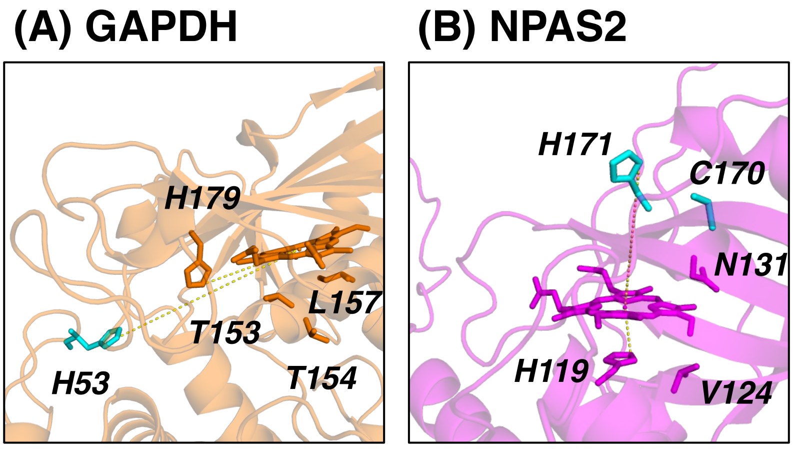

Fig. S3. (A) The predicted heme binding site for GAPDH (as in Fig. 4B) with His53 highlighted in cyan. The His179 (orange) Nε-Fe distance is 10.0 Å and the His53 Nε-Fe distance is 18.5 Å. The Nδ-Fe distances were longer than Nε-Fe distances in both cases, so these have not been shown. **(B)** The predicted heme binding site for NPAS2 (as in Fig. 5D) with Cys170/His171 highlighted in cyan. The His119 (magenta) Nε-Fe distance is 5.0 Å and the His171 (cyan) Nδ-Fe distance is 17.3 Å.

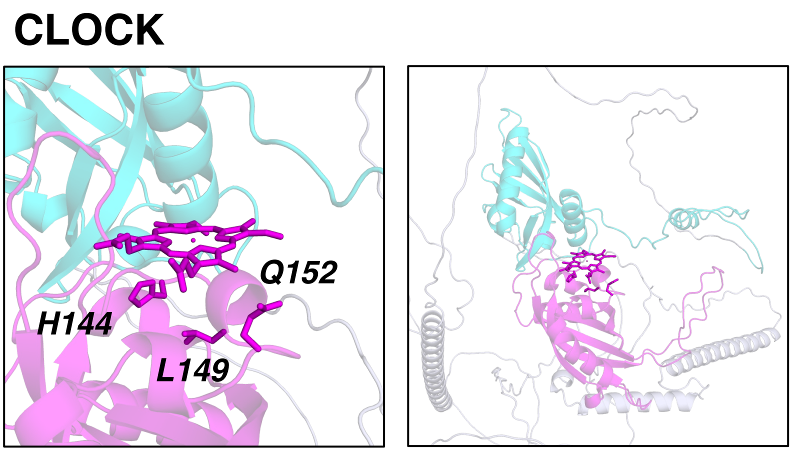

Fig. S4. The predicted heme binding site from Fig. 4A except this time the docking was performed in the AlphaFold model for CLOCK which contains both PAS-A (magenta) and PAS-B (cyan) domains. The rest of the protein is shown in grey. The left-hand panel shows the predicted heme binding site, and the right-hand panel shows the full length structure of the AlphaFold model with the predicted heme site for completeness. The PAS-B domain occupies the space above the heme which is consistent with Fig. 4A.
